# Synergic combination of the sol–gel method with dip coating for plasmonic devices

**DOI:** 10.3762/bjnano.6.52

**Published:** 2015-02-19

**Authors:** Cristiana Figus, Maddalena Patrini, Francesco Floris, Lucia Fornasari, Paola Pellacani, Gerardo Marchesini, Andrea Valsesia, Flavia Artizzu, Daniela Marongiu, Michele Saba, Franco Marabelli, Andrea Mura, Giovanni Bongiovanni, Francesco Quochi

**Affiliations:** 1 University of Cagliari, Department of Physics, S.P. Monserrato-Sestu Km 0.7, 09042 Monserrato, Italy; 2 University of Pavia, Department of Physics, Via Agostino Bassi 6, 27100 Pavia, Italy; 3 Plasmore S.r.l., Via Grazia Deledda 4, 21020 Ranco, Italy; 4 University of Cagliari, Department of Chemistry and Geology, S.P. Monserrato-Sestu Km 0.700, 09042 Monserrato, Cagliari, Italy

**Keywords:** biosensors, nanodevices, plasmonics, sol–gel, thin films

## Abstract

Biosensing technologies based on plasmonic nanostructures have recently attracted significant attention due to their small dimensions, low-cost and high sensitivity but are often limited in terms of affinity, selectivity and stability. Consequently, several methods have been employed to functionalize plasmonic surfaces used for detection in order to increase their stability. Herein, a plasmonic surface was modified through a controlled, silica platform, which enables the improvement of the plasmonic-based sensor functionality. The key processing parameters that allow for the fine-tuning of the silica layer thickness on the plasmonic structure were studied. Control of the silica coating thickness was achieved through a combined approach involving sol–gel and dip-coating techniques. The silica films were characterized using spectroscopic ellipsometry, contact angle measurements, atomic force microscopy and dispersive spectroscopy. The effect of the use of silica layers on the optical properties of the plasmonic structures was evaluated. The obtained results show that the silica coating enables surface protection of the plasmonic structures, preserving their stability for an extended time and inducing a suitable reduction of the regeneration time of the chip.

## Introduction

Plasmonic nanostructures have gained increasing attention for their surface plasmon resonance (SPR)-related properties, which can be exploited in innovative technological applications. SPR is a phenomena arising from the interaction between the incident electromagnetic radiation and the conduction electrons present on a metal surface. Such coupling leads to an enhancement and a spatial confinement of the electromagnetic field at a metal–dielectric interface [1–5]. Recently, due to such remarkable properties, biosensing technologies based on plasmonic nanostructures have attracted significant attention, particularly in the development of label-free sensors [6–8]. It is well known that surface plasmons (SPs) are extremely sensitive to the refractive index of the dielectric medium [1–2,9] and the two plasmons modes, surface plasmon polaritons (SPPs) and localized surface plasmons (LSPs), can be used for sensor applications [8,10–11].

However, in order to use this technology for sensing of a specific analyte, plasmonic-based devices require modifications of the metal surface that exceed some limitations of a bare metal surface [7–8]. The type of surface modification depends strongly on the application of the materials and can be achieved by various approaches [7–8,11–12]. Surface modification serves to stabilize the sensing platform (as in the case of metal nanoparticles) and provides a specific affinity, resulting in improved selectivity [7–8,11–12]. Furthermore, the surface chemistry of thiol-based self-assembled monolayers has shown some limitations mainly related to their temporal stability [13–14]. Therefore, research has been focus on the development of an ideal combination of surface functionalization methods and effective plasmonic structures for the detection and/or recognition of specific analytes.

However, independent from the considerations of the final application, sensing requires a chemically stable and optically tunable, dielectric platform, which should be properly functionalized: given these requirements, the silica layer coating represents a highly suitable method. However, the insertion of a silica layer between a plasmonic metal surface and a target molecule is not trivial. In fact, the electromagnetic field strength at a metal–dielectric interface decays exponentially from the metal surface [1–2,9]. Therefore, the proper deposition of a silica layer on the plasmonic structure is a critical factor since it can drastically reduce the action of the plasmonic field, due to the resulting increase in the distance from the metal surface. On the other hand, as demonstrated in our previous works, when the thickness of the silica layer is carefully controlled, a spatial redistribution with a consequent enhancement of the plasmonic field can be achieved [15–16]. However, a fine control of the layer thickness is also very important for plasmonic–photonic coupled devices [10,16–19].

For this purpose, the use of the sol–gel approach combined with the dip-coating technique to produce a silica layer is a suitable method for the modification of a plasmonic surface. In addition to preserving the nanostructured surface shape and its plasmonic action, this approach presents a proper platform for specific chemical functionalizations and, furthermore, it allows embedding of proper fluorophores and/or also specific molecules [20–22].

As previously mentioned a fine control of the layer thickness and of the matrix properties is critical. In this sense, many parameters affect the sol–gel mechanism reaction [23–26] and, therefore, the sol–gel process and the deposition technique should be optimized. To the best of our knowledge, few examples related to the use of a sol–gel approach for coating a planar nanostructure have been proposed in the literature [15–17,27]. Yet none of these works presented a comprehensive investigation of the effect of the key processing parameters.

The key processing parameters that allow for the fine-tuning of the silica layer thickness on a plasmonic structure were studied in this work. The plasmonic nanostructures were coated with conformal silica layers of controlled thickness using an optimized, combined sol–gel/dip-coating technique. The effects of the silica layer on the optical properties of the plasmonic nanostructure for sensing applications were investigated.

## Results and Discussion

### Silica coating control

In order to investigate the effect of the silica layer coating on the optical response of the plasmonic structure, the first step is to understand the role that the main parameters play on the layer thickness. Thus, the first goal was to prepare silica reference films and monitor their thickness as a function of various processing parameters, such as: pH, solvent concentration, aging time and withdrawal speed. These parameters play a significant role in the sol–gel mechanism reaction, affecting the microstructure homogeneity and the film thickness [23–27]. Moreover, a detailed optical study of the silica-coated plasmonic structure was performed.

**Scheme 1:**
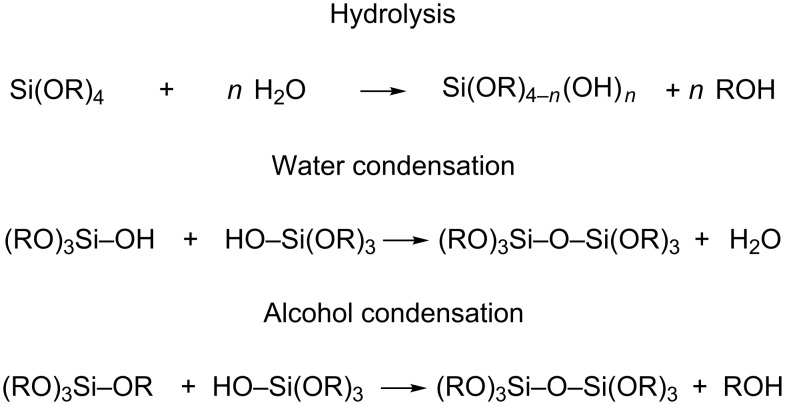
Simplified sol–gel mechanism reaction for a tetraalkoxysilane [23].

The sol was prepared using tetraethoxysilane (TEOS) as a silica precursor, water at pH 1, 2, 3, 4 and 5, HCl (as a catalyst) and ethanol (EtOH). A water:TEOS molar ratio of five was used. The films were produced by dip-coating onto glass microscope slides with withdrawal speeds ranging from 0.6 to 1000 mm/min. The coated films were dried for 48 h at room temperature under ambient conditions. The thickness, refractive index and uniformity were evaluated and spectroscopic ellipsometry (SE) was employed as the main characterization technique to evaluate the film thickness. The film thickness was monitored as a function of different processing parameters, namely, pH, aging time, EtOH dilution, and withdrawal speed. The sol–gel process using silicon alkoxide precursors involves hydrolysis and condensation reactions, which lead to the formation of a silica network (Scheme 1) [23].

Many processing parameters can affect the rate of hydrolysis and condensation, and thus, the features of the final material. Previous studies have reported the dependence of the sol–gel film thickness on the withdrawal speed, water:precursor molar ratio, and aging time [26,28–31]. However, all processing parameters must be controlled to carefully optimize the deposition onto the plasmonic nanostructures. In this study, a molar ratio of approximately 5 was used for all sols to favor a more complete hydrolysis and to produce more stable films [23,28–31].

**Figure 1:**
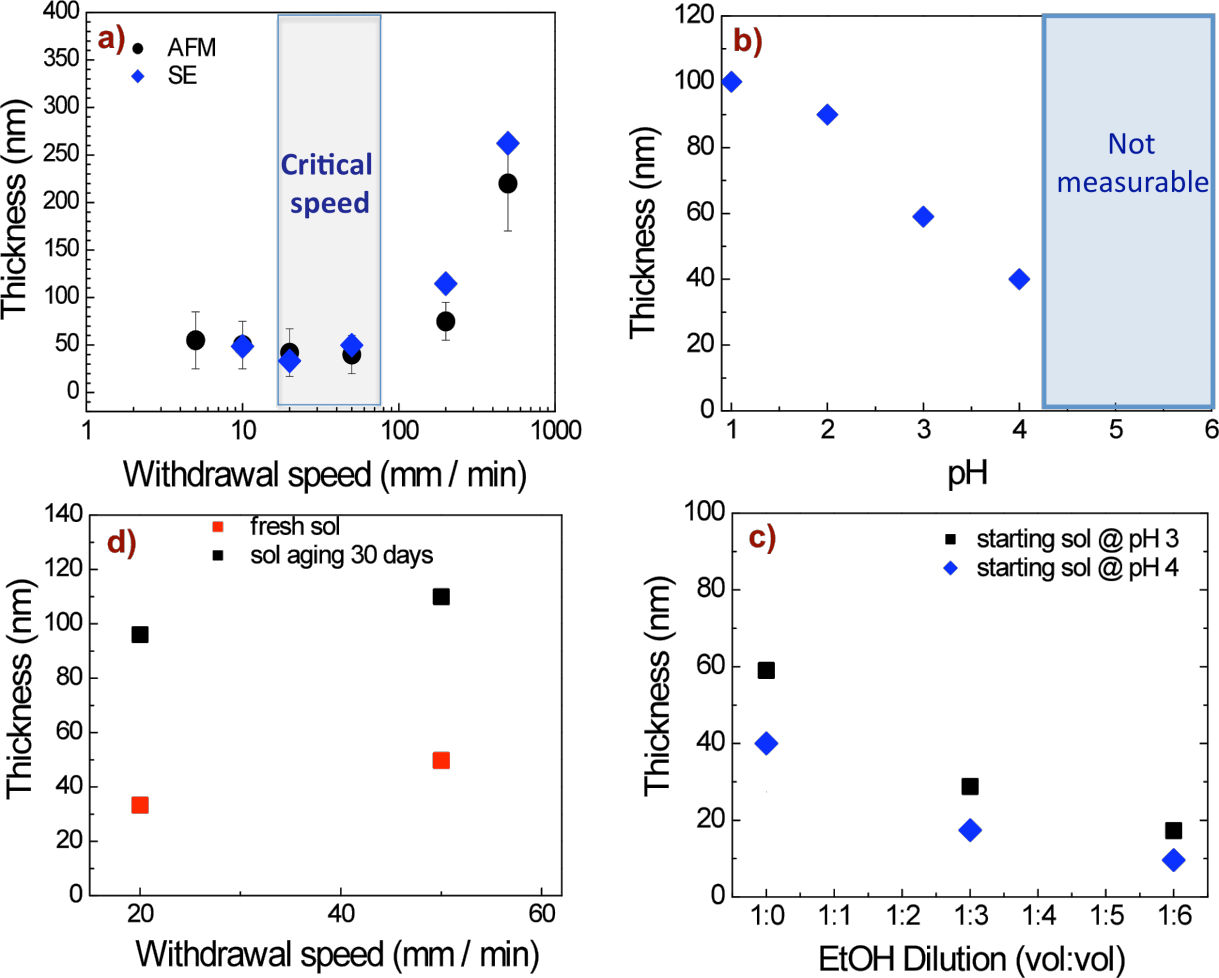
(a) Film thickness as a function of withdrawal speed for a sol of pH ≈4 and few days of aging, comparing results from both AFM and SE measurements. (b) Plot of the thickness versus starting pH value obtained at a withdrawal speed of 50 mm/min. (c) Plot of the thickness versus EtOH dilution at a withdrawal speed of 50 mm/min. (d) Plot of the thickness of a film prepared from fresh sol (starting pH ≈4) and from sol after 30 days.

Figure 1 shows the film thickness as a function of different processing parameters. Figure 1a shows a plot of the film thickness as a function of the withdrawal speed for films deposited on a bare glass substrate using a sol of starting pH ≈4 and few days of aging. In a matter similar to that previously observed by Faustini et al. [26], a critical withdrawal speed (≈50 mm/min) can be observed at which a minimum film thickness is achieved. In this regard, we note that the resulting films prepared at the critical speed of ≈50 mm/min are homogeneous, transparent and crack-free. The film thickness was also estimated using AFM with good agreement with the SE measurements.

The film thickness as a function of starting pH was also monitored. Figure 1b reports the film thickness obtained by keeping the withdrawal speed fixed (50 mm/min), while using different starting pH values. A decrease in the thickness with increasing pH (until pH 4) can be observed. These results highlight that film thickness is strongly affected by pH. From the literature [28–31] it is known that the pH value affects the hydrolysis and condensation processes, and thus, it influences the morphology and structure of the resulting final material.

The film thickness, as expected [28–31] was also affected by aging time and dilution of the coating sol. Figure 1c,d shows the thickness of films prepared at a fixed withdrawal speed (50 mm/min) as a function of aging time (starting pH ≈4) and ethanol dilution (for two different starting pH values). The thickness decreases with the ethanol dilution (Figure 1c) and increases with the sol aging time (Figure 1d). According to the literature [26–29], the thickness is proportional to the sol viscosity; accordingly, it can be reduced by increasing the dilution since, which decreases the viscosity of the sol. On the other hand, when increasing the aging time, the viscosity increases, resulting in an increased thickness [23,28].

From the above data, the sol pH and dilution seem to have stronger effects on film thickness. These results were used as a calibration method to control the silica layer deposition on plasmonic structures. The film thickness was carefully tuned by controlling the pH and increasing the EtOH dilution of the sol, while operating at critical withdrawal speed. Specifically, for films prepared from fresh sol of pH ≈4 and 1:6 (v/v) EtOH dilution, a thickness less than 10 nm (Figure 1b,c) was reached. Therefore, the thickness of the silica films was controllable on the nanometer scale.

**Figure 2:**
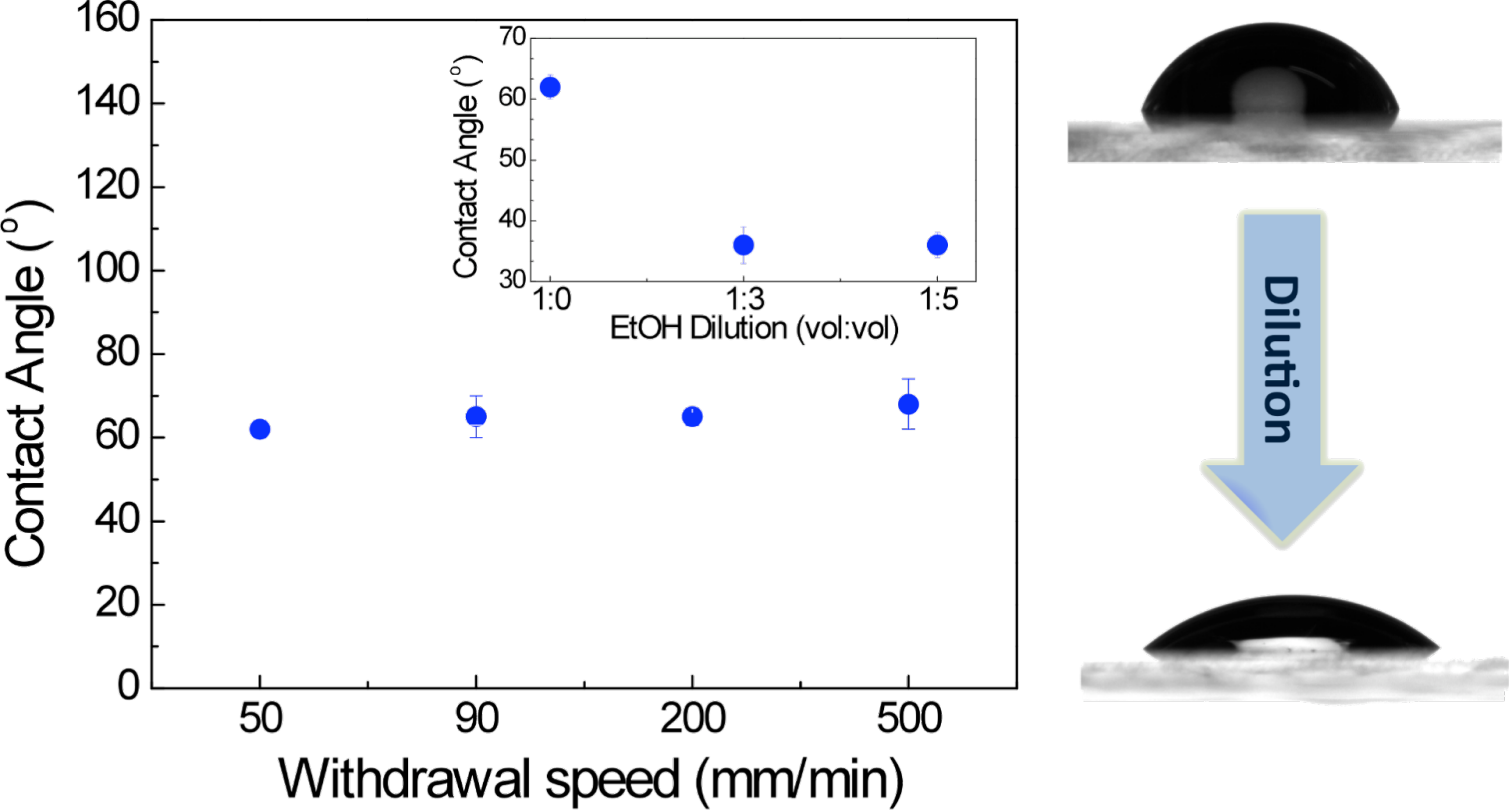
Contact angle measurements of a silica layer deposited by dip coating onto a glass substrate at different withdrawal speeds (left). Contact angle measurements of a silica layer deposited by dip coating at a 50 mm/min withdrawal speed onto a glass substrate as a function of ethanol dilution of the initial sol (right).

The control of the hydrophilic or hydrophobic property of the surface is an important characteristic from the perspective of biosensing applications since it also allows for altering the surface affinity for specific molecules. The surface of the films prepared from fresh sol of pH ≈4 was further characterized through contact angle measurements. As shown in Figure 2, these results highlight that the films are hydrophilic with a contact angle of 65°. This value is independent of the film thickness, but decreases with ethanol dilution up to 34° (inset of Figure 2). This suggests a correlation between the distribution of hydroxy groups, which affects film wettability, and the sol dilution. This change in the contact angle is probably due to the modification of the silica microstructure induced by an increase of the ethanol concentration, which is also supported by the decrease of the refractive index values for films prepared from diluted sols [28–31].

**Figure 3:**
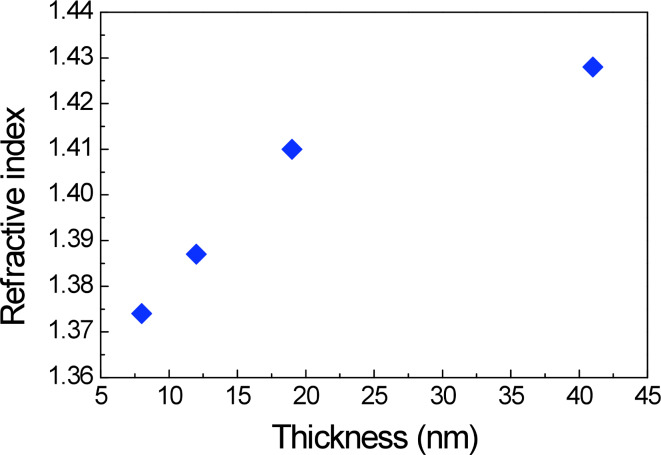
Refractive index, *n*, at 750 nm, evaluated by spectroscopic ellipsometry of silica layers deposited onto a glass substrate at a fixed withdrawal speed (50 mm/min) for different EtOH dilution (v/v = 1:0, 1:2, 1:4, 1:6).

Figure 3 displays the refractive index, *n*, at the intermediate wavelength of 750 nm for silica layers of different thickness. The films were deposited onto a glass substrate at a fixed withdrawal speed (50 mm/s) for different EtOH dilutions (v/v = 1:0, 1:2, 1:4, 1:6) evaluated by SE.

Furthermore, the simultaneous analysis of SE data and transmittance spectra in the vis–NIR region were used to obtain the optical characteristics (*n* and the extinction coefficient, *k*) and to confirm the optical quality of the silica layers [15]. In particular, the SE measurements also indicate that the extinction coefficient is negligible through all the visible (vis) and near-infrared (NIR) range.

### Coating of plasmonic structure

**Figure 4:**
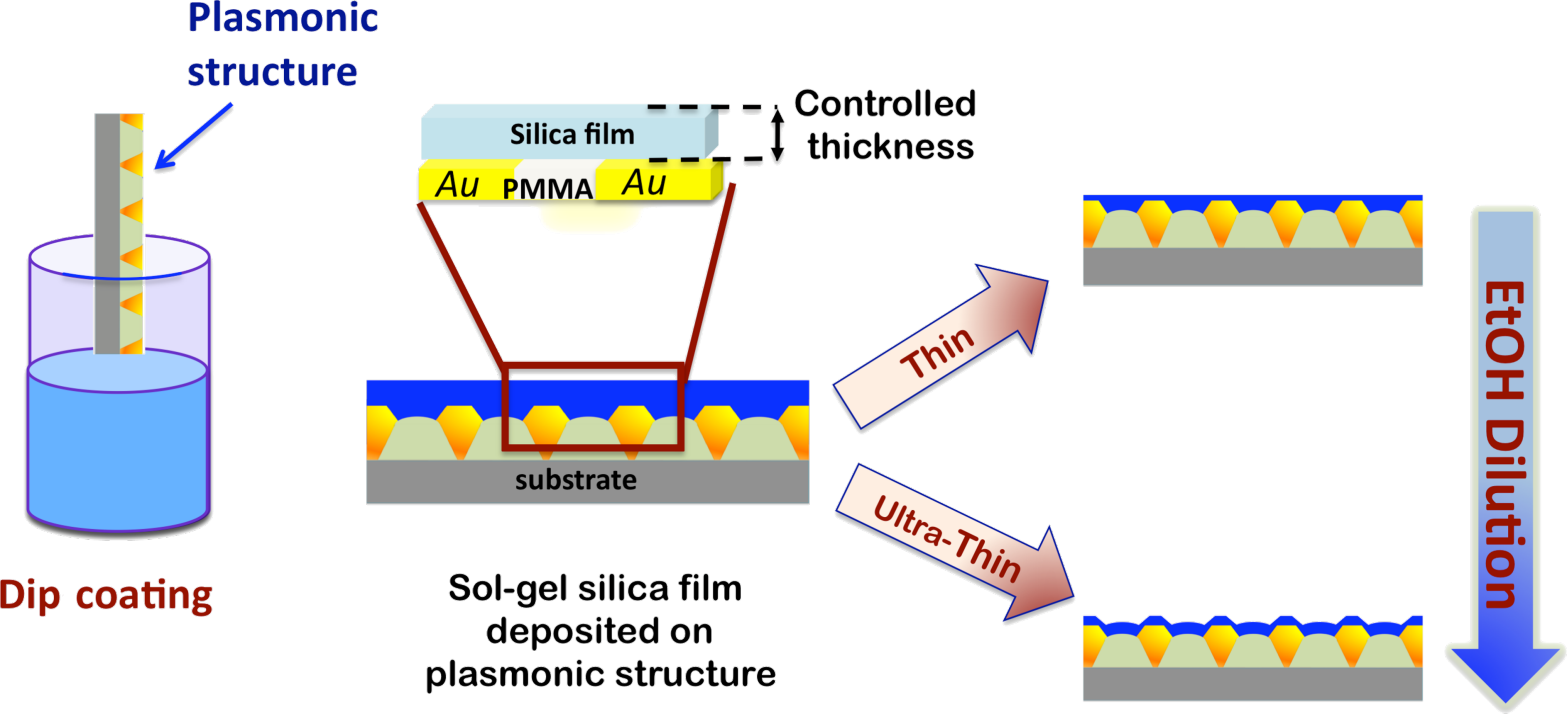
Tunable-thickness silica layer deposition onto plasmonic structures by dip coating. The thickness control of the layer is achieved by controlling pH, sol aging, withdrawal speed and the EtOH concentration.

The results of the previous section were used as a calibration method to properly coat the surface of plasmonic devices consisting of a 2D array of truncated conical poly(methyl methacrylate) (PMMA) pillars [32–34]. Such a plasmonic platform has been shown to work efficiently as an SPR-sensitive surface for biosensing applications in medical diagnostics [35]. A sketch of the pillar profile along a normal cross section is provided in Figure 4, where the procedure was adapted for silica layer deposition as schematically represented. The plasmonic structure is dipped in the sol and then withdrawn at a constant rate.

**Figure 5:**
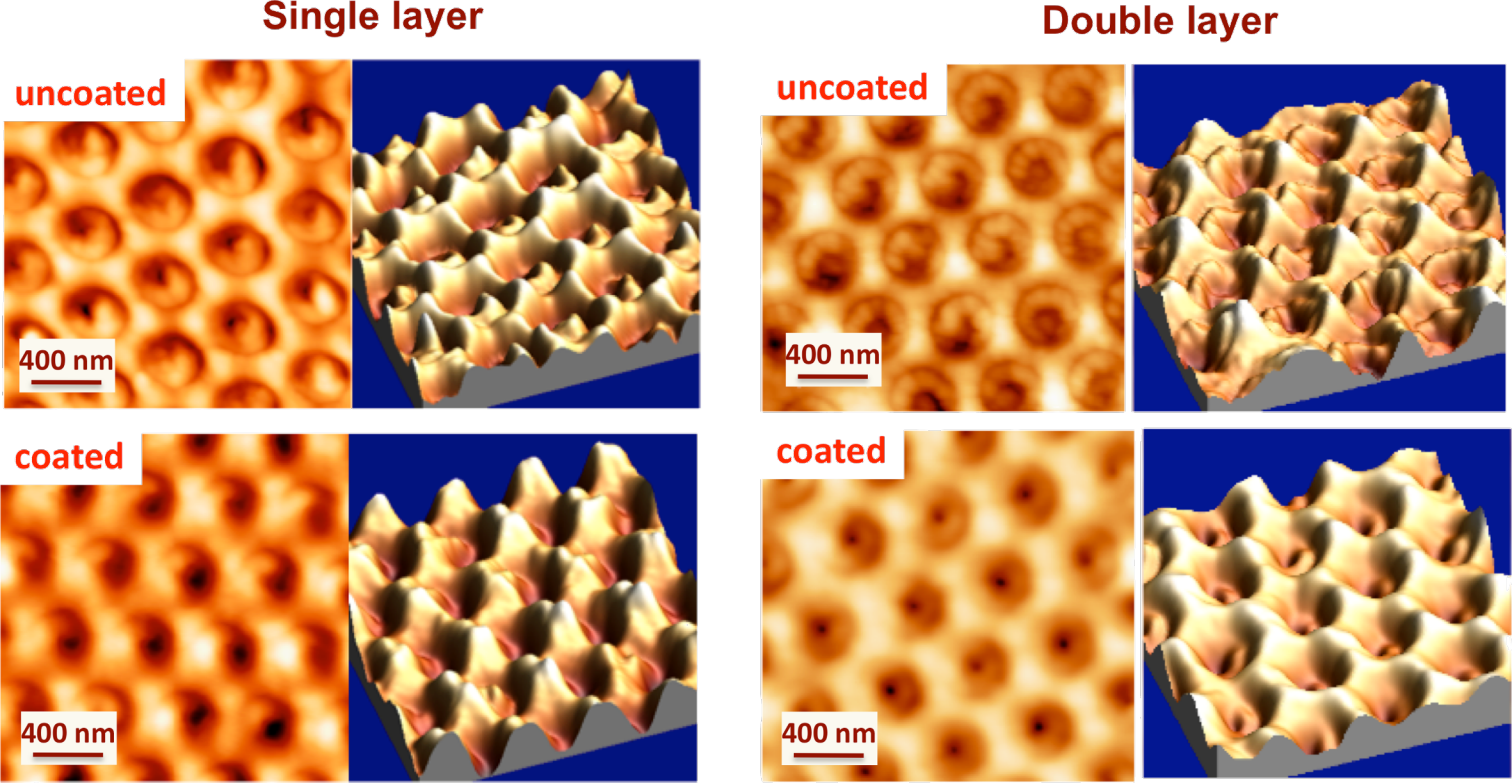
AFM topographies (semi-contact mode) and 3D morphological reconstruction of typical plasmonic structures before and after coating with a single layer of ≈6 nm (left) and a double layer (right) of silica. The coatings were realized by using 1:6 (v/v) EtOH dilution of the starting sol (pH ≈4) at a withdrawal speed of 50 mm/min.

Figure 5 shows AFM topographies (performed in semi-contact mode) of the plasmonic structure before and after coating with a single layer of ≈6 nm and a double layer. The coatings were realized by using 1:6 (v/v) EtOH dilution of the starting sol (pH ≈4) at a withdrawal speed of 50 mm/min. The single layers were deposited by a single dip and the double layers were deposited by two dips (where the second layer was deposited 60 s after the first layer).

Conformal silica layers (i.e., those which maintain the original shape of the nanostructured surface) with a homogeneous coating was achieved, as is evidenced by the AFM topography after coating (Figure 5). The AFM images of the surface reported in Figure 5 show the topography and the 3D morphological reconstruction of typical plasmonic structures before and after coating. A regular deposition of the pillars, with a hexagonal lattice structure, is clearly observed. Similar images were obtained for samples coated with layers of different thicknesses.

### Effects of silica layer on plasmonic structures

**Figure 6:**
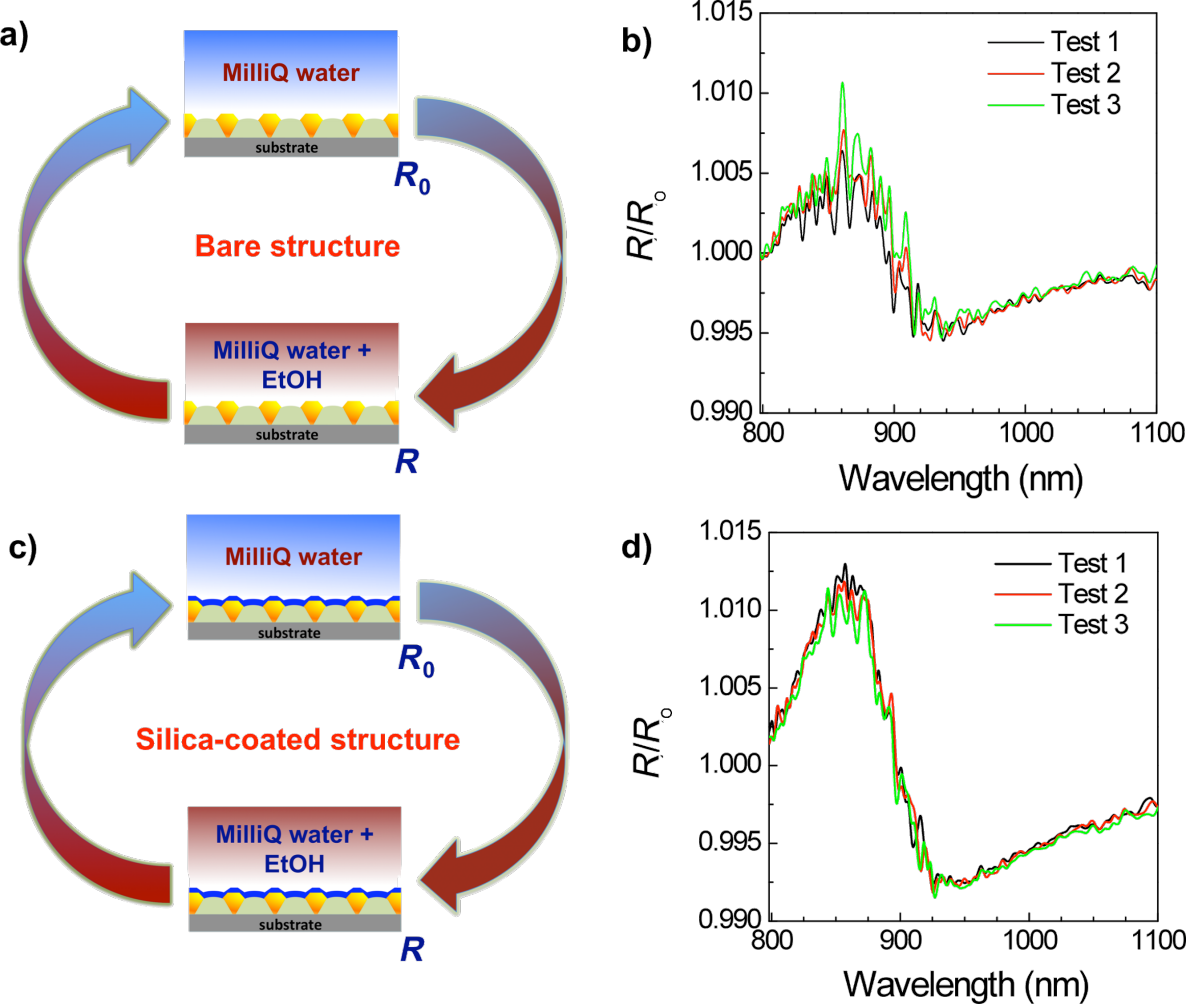
Simplified schemes showing the procedure used to perform the optical sensing and chip regeneration tests for (a) bare (uncoated) and (c) silica-coated structures. Normalized reflectance spectra (*R*/*R*_0_) of a plasmonic, nanostructured chip for the (b) bare (uncoated) chip, tested with 12 h regeneration time and (d) an ≈6 nm thick silica layer coated chip, tested with 30 min regeneration times.

The effect of using a silica-coated, plasmonic surface (optimized for biosensing) was analyzed by comparing the optical response before and after the coating process. The effects resulting from silica coating were assessed through the direct measurement of the sensitivity of the plasmonic structures to refractive index changes in aqueous solution. In the reflectance spectra, the plasmonic absorption feature is shifted to longer wavelengths due to the modiﬁcation of the dielectric function surrounding the gold [1–4,15–17]. Figure 6 reports a simplified scheme showing the procedure used to perform the optical sensing test and to regenerate the sensor chip. The normalized reflectance spectra (*R*/*R*_0_) of both a bare (uncoated), plasmonic, nanostructured chip and a chip coated with an ≈6 nm thick silica layer are shown. The reference reflectance spectrum (*R*_0_, Figure 6a) is measured with the chip immersed in MilliQ water, and the reflectance spectrum (*R*, Figure 6c) is measured with the chip immersed in MilliQ water and EtOH (concentration, 3%). The optical response of the system was monitored through a series of measurements on the same solution (Figure 6, right) by evaluating the relative reflectance spectra (*R*/*R*_0_) of the plasmonic, nanostructured surface coated with an ≈6 nm thick silica layer versus time. These results show that the use of silica layers leads to an improvement of the plasmonic surface protection. In particular, an easy reversibility and the reduction of the regeneration time of the chip (from 12 h to 30 min) was observed. Such a result is particularly interesting for the development of efficient, real-time sensors. Furthermore, we observe a clear improvement of the device sensitivity with respect to refractive index variations [15].

Moreover, the resulting silica sol–gel thin film behaved as a host matrix in which suitable vis or NIR fluorophore dopants could be dispersed. This is useful to couple the plasmonic and the emission signals, thus preventing undesirable metal-induced radiative emission quenching [17]. Finally, using organically modified alkoxides, the silica platform can be directly functionalized with selected functional groups required for specific detection schemes, all in a single-step process.

## Conclusion

In this work, a combined optimized sol–gel method/dip-coating technique was exploited as a promising low-cost method for the realization of a silica-coated plasmonic platform with a high degree of silica thickness control. The key processing parameters that allow a fine-tuning of the silica layer thickness were investigated. This method enables modification of the nanostructure surface, while preserving the plasmonic functionality and broadening the potential of the plasmonic-based sensing applications. Indeed, such a sol–gel layer represents a proper framework to protect the plasmonic surface against external agents and reduces the sensor chip regeneration time. In addition, it represents a suitable platform to selectively bind target molecules with specific chemical functionalities through proper functionalization.

## Experimental

Tetraethoxysilane (TEOS) with purity >99%, absolute ethanol (EtOH) with purity >99%, acetonitrile (MeCN) with purity >99% and distillated water (H_2_O) were used as received without further purification. All reagents and solvents were purchased from Sigma-Aldrich. The sol precursor was prepared by mixing TEOS (8 mL), EtOH (20 mL) and H_2_O (3.75 mL) under stirring at room temperature (RT) for 24 h. Subsequently, a volume of 6 mL of EtOH/MeCN (v/v = 1:1), and HCl (until pH 1, 2, 3, and 4 was reached) were added to 9 mL of the sol and the mixture was maintained under stirring at 50 °C for 1 h.

Additional diluted solutions were prepared by mixing a fresh TEOS solution with different amounts of EtOH (v/v = 1:0, 1:1, 1:2, 1:3, 1:4, 1:5, 1:6). The resulting diluted TEOS solutions were gently mixed in a closed vessel at RT. The molar ratio of water:precursor of the starting solution was 5 for all sols.

Soda-lime glass was employed as a substrate for reference film deposition. Before coating, the substrates were cleaned with water and soap, distilled water, acetone and finally, rinsed with isopropanol.

The films were deposited by dip coating at withdrawal speeds ranging from 0.01 to 20 mm/s. The glass substrates were immersed into the sol at a constant dipping speed of 8 mm/s and kept inside the bath for 25 s. After withdrawal, all films were dried at room temperature for 48 h.

The same method was used for coating the plasmonic structures. The single layers were deposited with one dip and the double layers were deposited with two dips (where the second layer was deposited after 60 s after the first layer).

The plasmonic structures were developed by Plasmore S.r.l. using colloidal lithography. They consisted of a 2D array of truncated, conical poly(methyl methacrylate) (PMMA) pillars (with a height of about 150 nm and a diameter of 350 nm) embedded into a gold layer that was used to fill the space between the pillars, resulting in a perforated-like film [32–35] .

An ND-R rotatory coater (Nadetech Innovations) was used for the deposition of all silica films.

An NT-MDT Solver-Pro AFM was used to analyze the topography and to estimate the thickness of the films. The measurements were performed at a scan speed of 0.5–1 Hz in semi-contact mode. In order to evaluate the film thickness using AFM, the fresh films deposited on the glass substrate were cut with a scalpel. After 48 h at room temperature, this cut on the film was observed by AFM for the thickness estimation. The evaluation of the surface roughness and thickness was performed by using WSxM 5.0 Develop3.2 software.

The wettability of the silica ﬁlms was measured by using a Dataphysics Contact Angle System OCA, where a sessile drop method was used to measure the contact angle of a 5 μL distilled water droplet, which was applied to the surface by means of a syringe.

A variable angle SE (VASE, J. A. Woollam Co., Inc.) in the 0.25–2.5 μm wavelength range was used for the SE analysis, and an Agilent Cary 6000i spectrophotometer was used for normal-incidence reflectance and transmittance measurements in the 0.2–1.6 μm range.

The SE, reflectance and transmittance spectra were analyzed using dedicated WVASE32^®^ software. Angle-resolved reflectance measurements were performed in the spectral range between 0.5 and 1.2 μm on the bare and coated plasmonic samples. This was made possible by a custom-built micro-reflectometer setup coupled to an FTIR (Bruker, IFS66).
